# Risk analysis and clinical outcomes of intraoperative periprosthetic fractures: a retrospective study of 481 bipolar hemiarthroplasties

**DOI:** 10.1186/s13018-019-1494-1

**Published:** 2019-12-11

**Authors:** Petri Bellova, Hinnerk Baecker, Sebastian Lotzien, Marvin Brandt, Thomas A. Schildhauer, Jan Gessmann

**Affiliations:** 1 Department of Orthopedic and Trauma Surgery, BG University Clinic Bergmannsheil Bochum, Bürkle-de-la-Camp Platz 1, 44789 Bochum, Germany; 2 Ruhr-University Bochum, Bochum, Germany

**Keywords:** Femoral neck fracture, Hemiarthroplasty, Intraoperative fracture, Risk factor, Femur shape, Dorr, mobility, Decision-making

## Abstract

**Background:**

Intraoperative periprosthetic fractures (IPF) are a well-described complication following hip hemiarthroplasty. Our aims were to identify risk factors that characterize IPF and to investigate postoperative mobility.

**Methods:**

We retrospectively reviewed 481 bipolar hemiarthroplasties for displaced femoral neck fractures; of which, 421 (87.5%) were performed without cement, from January 2013 to March 2018. Data on the patients’ demographics, comorbidities, femoral canal geometry (Dorr canal type, Canal Flare Index), surgeon’s experience (junior vs. senior surgeon), and timing of surgery (daytime vs. on-call duty) were obtained. In patients with intraoperative fractures, further information was obtained. Patient mobility was assessed using matched-pair analysis. Mobility was classified according to the NHFD mobility score. The chi-square test, Fisher’s exact test, and Fisher-Freeman-Halton exact test were used for comparison between categorical variables, while the Mann-Whitney *U* test was used for continuous variables. The data analysis was performed using SPSS.

**Results:**

Of 481 procedures, 34 (7.1%) IPFs were encountered. The Dorr canal type C was identified as a significant risk factor (*p* = .004). Other risk factors included female sex (OR 2.30, 95% CI .872–6.079), stovepipe femur (OR 1.749, 95% CI .823–3.713), junior surgeon (OR 1.204, 95% CI .596–2.432), and on-call-duty surgery (OR 1.471, 95% CI .711–3.046), although none showed a significant difference. Of 34 IPFs, 25 (73.5%) were classified as Vancouver type A. The treatment of choice was cerclage wiring. Within the 12 matched pairs identified, the postoperative mobility was slightly worse for the IPF group (delta = .41).

**Conclusions:**

IPF is a serious complication with bipolar hemiarthroplasty. The identification of risk factors preoperatively, in particular femur shape, is crucial and should be incorporated into the decision-making process.

## Background

Life expectancy is increasing worldwide, as is the proportion of older people in the overall population. Associated with increasing age is a decrease in bone mineral density, as well as muscle mass and strength, increasing the risk of falls and fall-related injuries. Approximately 95% of hip fractures are caused by falls from standing height [1, 2].

Fractures of the proximal femur are responsible for the largest use of resources for orthopaedic trauma in the world. In 2000, there were an estimated 424,000 hip fractures worldwide in men and 1,098,000 in women [3], and the incidence is projected to rise to 4.5 million by 2050 [4, 5]. Hip fracture in the elderly is associated with nearly 18–33% mortality within the first year after fracture [6]. There is also a considerable decrease in mobility. A third of the patients have a long-term decrease in daily activities and functionality that reduce their independence [6, 7].

The treatment of hip fractures can be either surgical or nonsurgical. Surgical treatment has been shown to be more cost-effective and provide lower complication rates, lower mortality, and improved rehabilitation when compared with nonsurgical treatment [8–10]. The choice of treatment is dependent on the fracture pattern, patient age, and comorbidities, as well as the availability of resources. For displaced femoral neck fractures (AO 31-B), hemiarthroplasty is the widely accepted treatment of choice in elderly patients [11].

Both cemented and uncemented hemiarthroplasties have been used successfully in the past. Functional results, complication rates, and mortality have been similar in randomized controlled trials [12–14]. The UK National Institute for Health and Care Excellence (NICE) guidelines, however, have clearly recommended the use of cemented implants in surgery with arthroplasty [15].

Complications following hip fracture surgery can either be medical or directly related to the surgery itself [16]. A widely known surgery-related complication with uncemented hip hemiarthroplasty is intraoperative periprosthetic fracture (IPF). IPF can be classified according to the Vancouver classification. A type A fracture involves the trochanteric area, a type B fracture the shaft area around the prosthesis, and a type C fracture occurs distal to the tip of the prosthesis. Each type is further subclassified as subtype 1 if there is only a cortical perforation, subtype 2 if there is a nondisplaced crack, and subtype 3 if there is a displaced unstable fracture pattern [17, 18]. In recent studies, the incidence of intraoperative fracture was shown to be significantly higher for uncemented compared with cemented hemiarthroplasty [9, 14, 19].

Specific risk factors have been outlined for the occurrence of these types of fractures. The type of fixation (cemented vs. uncemented) and Dorr canal type are known to be significant risk factors [20, 21]. Female sex has been shown to be a significant risk factor with total hip arthroplasty [22]. Our primary aim was to perform a risk analysis based on these risk factors, as well as on the timing of surgery (daytime vs. on-call duty) and the surgeon’s experience (junior surgeon vs. senior surgeon).

Our secondary aim was to classify and characterize all IPFs and summarize the different treatments performed. If follow-up radiographs were available, bony union was evaluated.

Our tertiary aim was to assess the postoperative mobility of patients who had sustained IPFs when compared with patients who had not. Little is known about the functional outcomes following an IPF, especially with bipolar hemiarthroplasty. Inadequate fixation of an IPF may lead to fracture displacement or non-union, persistent thigh pain, poor bone ingrowth, and aseptic loosening of the femoral stem [23]. These factors may lead to a significant decrease in function and mobility for patients. Along with this, secondary complications such as thrombosis, pneumonia, and death may be more likely.

## Patients and methods

Patients treated with a bipolar hemiarthroplasty from January 2013 to March 2018 following femoral neck fracture were included in this study and were retrospectively reviewed. Patients were identified using the hospital’s diagnosis and operative code system. Electronic records and radiographs were used. Exclusion criteria were diagnoses other than femoral neck fracture, pathological fractures, treatment options other than bipolar hemiarthroplasty and incomplete data. The ethical review committee of Ruhr-University Bochum approved this study (18-6389).

Data on the patients’ demographics (age and sex), presence of comorbidities (hypertension, coronary artery disease, diabetes mellitus, end-stage kidney disease, and consumption of steroids), ASA score, duration of surgery, surgical approach, surgeon’s experience, timing of surgery (on-call duty vs. daytime surgery), and specific IPF information were obtained.

**Fig. 1:**
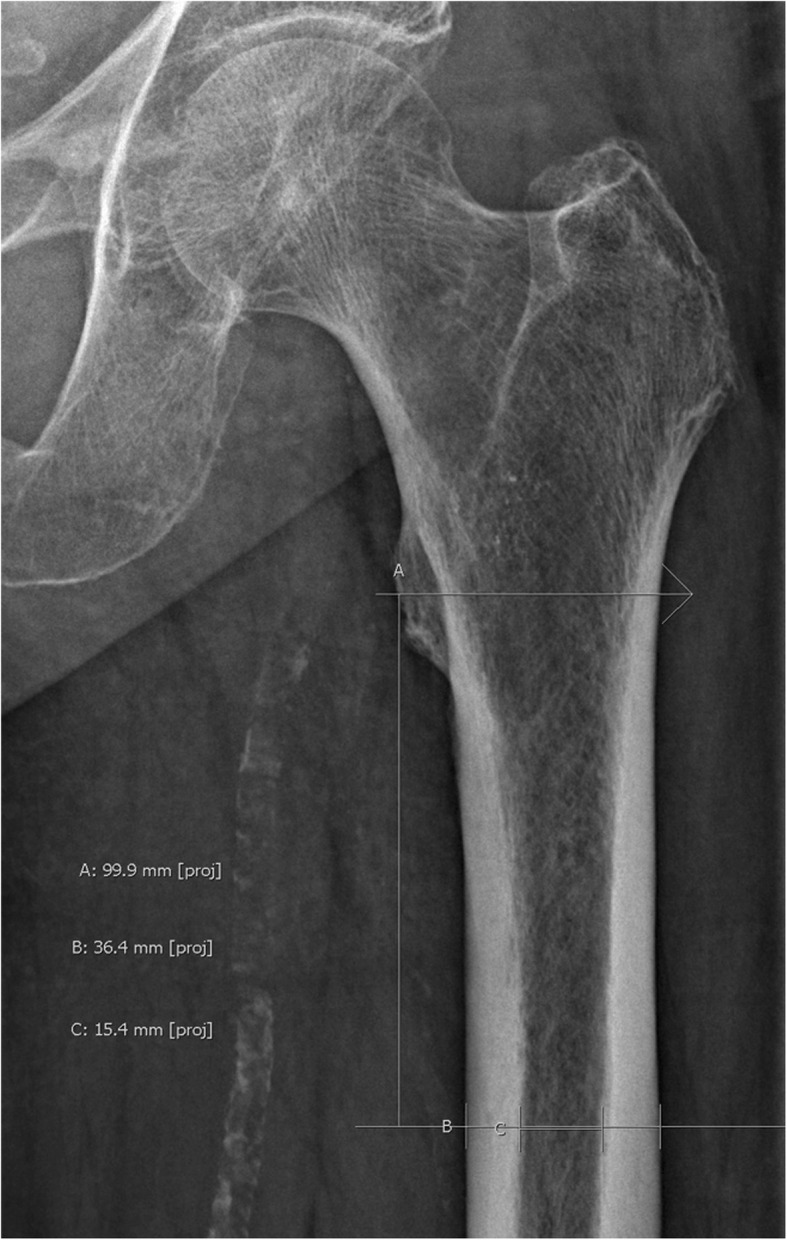
Measurement of mediolateral Cortical Thickness Index (CI). A represents the reference line perpendicular to the mechanical axis of the femur at the tip of the lesser trochanter. B represents a line 10 centimeters below and parallel to A. This line is limited by the periosteum of the femur. C represents the width of the medullary canal at the level of B. Mediolateral CI is then calculated as follows: (B − C)/C

Radiographs (preoperatively or postoperatively) of the patients were reviewed and analyzed by two independent authors (MB and SL) who were blinded to the characteristics of the patients. The patients’ femurs were classified according to the Cortical Thickness Index (CI) by Dorr et al. [24]. CI was defined as the ratio of cortical width minus endosteal width to cortical width at a level of 100 mm below the tip of the lesser trochanter on anteroposterior radiographs [25]. Higher values indicate thicker cortices. Type A exhibits thick cortices that begin at the distal end of the lesser trochanter and thicken quickly, producing a funnel shape and a narrow diaphyseal canal. Type B exhibits bone loss proximally and widening of the diaphyseal canal. Type C exhibits considerable loss of the thickness of the cortices resulting in a very wide intramedullary canal and a fuzzy appearance to the bone cortices [25] (Fig. 1). In arithmetical terms, a Dorr type A canal has an average mediolateral CI of .58, a type B of .50, and a type C of .42 [24]. Canal type was determined as a combination of CI and morphology.

Additionally, the Canal Flare Index (CFI) according to Noble et al. [26] was determined as the width of the femoral canal 20 mm above the mid-trochanteric line divided by the canal width at the isthmus. A CFI of less than 3.0 was described as a “stovepipe” (SP)-shaped canal, an index between 3.0 and 4.7 as “normal” (N), and an index above 4.7 as a “champagne flute” (CF)–shaped canal [27].

Due to the rotational deformity of the femur associated with fracture, measurements were taken on the contralateral femur. If not applicable, measurements were taken on postoperative radiographs.

Surgeons were subdivided into junior and senior surgeons. Senior surgeons were defined as having at least reached the consultant level. The timing of surgery was subdivided into daytime surgery and on-call duty surgery. Any procedure that started after 6 p.m. until 8 a.m. the following day, on weekends and on national holidays, was defined as on-call duty surgery, while any other procedure was defined as daytime surgery.

The duration of the surgery was recorded in minutes, and the types of anesthesia were divided into general anesthesia, regional anesthesia, or TIVA (total intravenous anesthesia).

In the presence of IPF, further information was obtained, including the steps leading up to the fracture, the location of the fracture, the respective treatment, and union. The steps leading up to the fracture were subdivided into (1) femoral canal preparation, (2) trial implant insertion and reduction, and (3) final implant insertion and reduction. The location of the IPF was classified according to the Vancouver classification for IPFs. Union could only be determined in the presence of available follow-up radiographs. Follow-up radiographs were only taken into consideration if taken at least 3 months postoperatively.

**Fig. 2:**
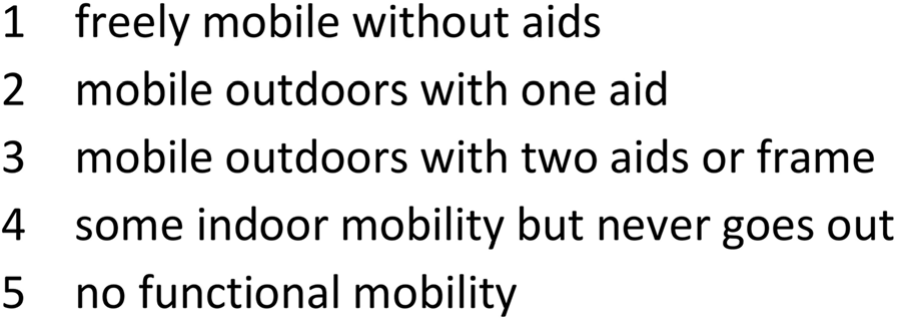
NHFD mobility score. The score comprises 5 levels of mobility with “1” representing the highest level and “5” the lowest level of mobility

The mobility of patients was investigated using the National Hip Fracture Database (NHFD) mobility score with 1 indicating the highest level of mobility and 5 indicating the lowest level of mobility (Fig. 2), which is counterintuitively higher for less mobile patients [28]. The preoperative and postoperative mobility of patients was assessed. Preoperative mobility was defined as pre-fracture mobility. Postoperative mobility was defined as the best level of mobility reached by patients postoperatively. If over the years the patients had become bedbound secondary to medical events, such as stroke, etc., this was not included in our documentation. Questionnaires were sent to the patients in the (intraoperative) fracture group first. If they were not returned within 4 weeks, patients or relatives were called. Numbers were extracted from our hospital database system. All respondents were included in a matched-pair analysis. Criteria for matching with patients from the non-fracture group were sex, age group (5-year spans) and time span of surgery ± 6 months. After matching the criteria mentioned, the procedure of sending questionnaires and phone calling was repeated with the corresponding patients identified in the non-fracture group. After data acquisition, all patients with different levels of preoperative mobility were excluded, including only patients with identical preoperative mobility eligible in the matched-pair analysis.

Data entry was performed using a spreadsheet application (Microsoft Excel, Microsoft Corp., Redmond, WA). Frequency tables and descriptive statistics were presented for all variables; categorical variables were presented as proportions, and continuous variables were presented as the mean. The chi-square test, Fisher’s exact test, and Fisher-Freeman-Halton exact test were used for comparison between categorical variables, while the Mann-Whitney *U* test was used for continuous variables. Odds ratios (OR) with 95% confidence intervals (CI) were presented for predetermined possible predictors of intraoperative fractures [21, 29]. The data analysis was performed using SPSS (SPSS Inc., Chicago, IL, version 25).

## Results

Between January 2013 and March 2018, we identified 481 patients who underwent bipolar hemiarthroplasty for femoral neck fracture. In total, 349 of 481 patients (72.6%) were female, and the average age was 83.7 years (range 54–103). The average duration of surgery was 75.4 min (range 28–166), while the average hospital stay was 11.6 days (range 1–116). A total 421 of the 481 procedures (87.5%) performed were without cement, while in 407 patients (84.6%), the Zweymüller Alloclassic shaft was implanted using a non-cemented technique. Of the 60 (12.5%) hemiarthroplasties with cement, 58 (12.1%) were performed using the Müller straight stem. A total 192 of 481 (39.9%) procedures were performed by junior surgeons, and 289 (60.1%) were performed by senior surgeons. There were 270 of 481 (56.1%) procedures performed during on-call duty, and 211 of 481 (43.9%) procedures were performed during the daytime.

**Fig. 3:**
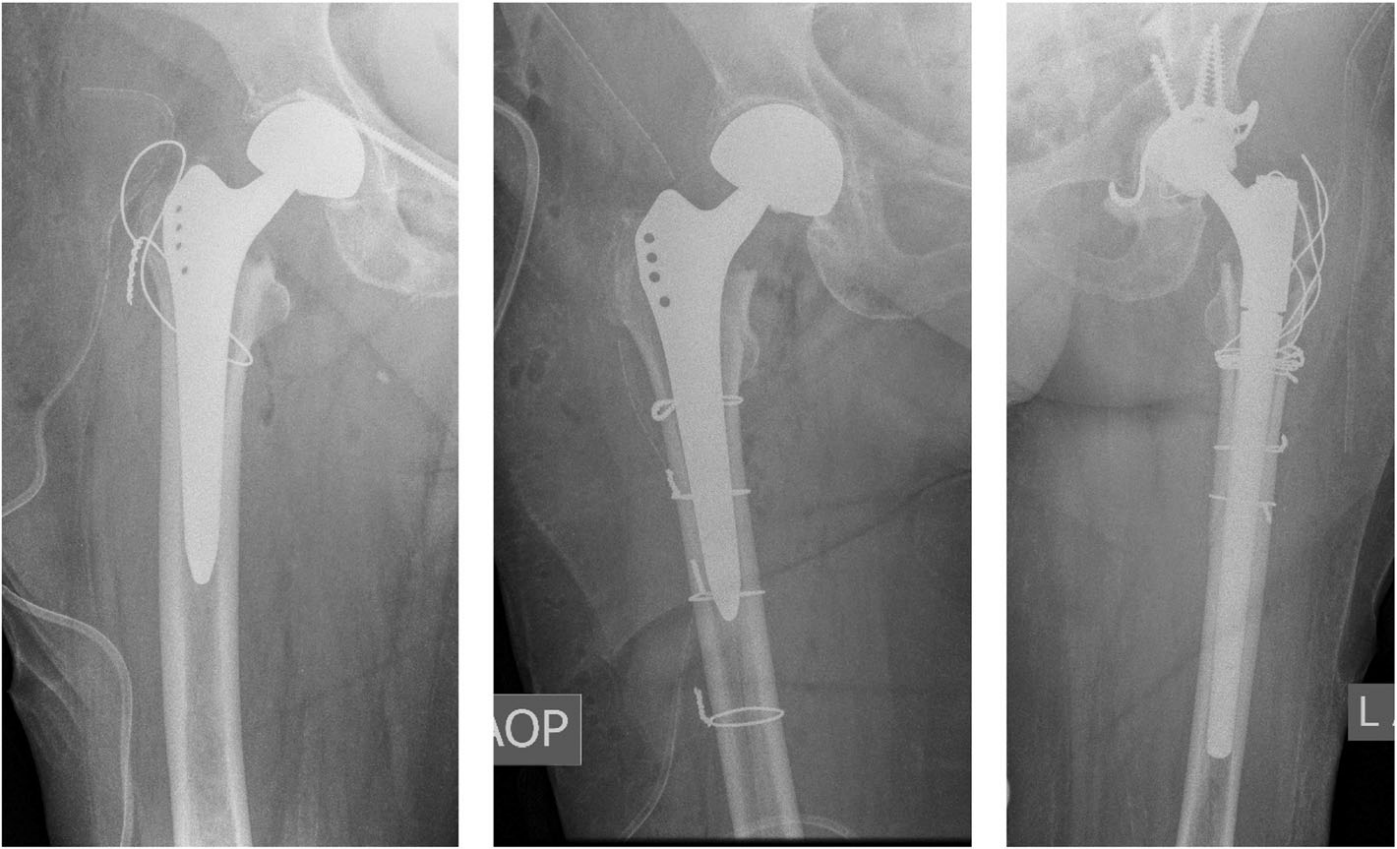
Salvage treatments for IPF. The panel labels depict the side shown on the radiographs. **a** Figure of 8 cerclage wire. **b** Single loop cerclage wires around shaft area. **c** Revision arthroplasty after postoperative implant failure following IPF

In total, 34 (7.1%) IPFs were identified; of which, 25 (73.5%) were classified as Vancouver type A, 8 (23.5%) as Vancouver type B, and 1 (2.9%) as Vancouver type C fractures. Thirty-three of the fractures occurred during cementless hemiarthroplasty, while a single fracture (2.9%) occurred during cemented hemiarthroplasty. In total, 33 IPFs were recorded in 421 uncemented arthroplasties (7.8%), while one IPF was recorded in 60 cemented arthroplasties (1.7%). In 29 of 34 IPFs, information on the timing of the fracture could be obtained. In 11 of 29 (37.9%) procedures, the fracture occurred during the femoral canal preparation (“1” = reaming, rasping, broaching), in 6 (20.7%) during the trial implant insertion and reduction (“2”), while in 12 (41.3%) procedures, the fracture occurred during the final implant insertion and reduction (“3”). In 21 (61.8%) patients, surgeons selected the transgluteal “Bauer” approach, whereas in 13 patients (38.2%), they chose the anterolateral “Watson-Jones” approach. Femur morphology was classified according to the Dorr classification [25] as well as by the Canal Flare Index (CFI). Four (11.8%) of the patients’ femurs that sustained an intraoperative fracture were classified as Dorr type A, 15 (44.3%) as Dorr type B, and 15 (44.3%) as Dorr type C. There was no CF femur recorded in the fracture group, along with 23 (67.6%) N and 11 (32.4%) SP femurs. The treatment instituted in the event of an intraoperative fracture depended on the type of fracture. In 23 of 34 patients, a double loop cerclage wire fixation around the proximal femur and the abductor musculature tendons was implemented; in one patient, this type of fixation was combined with conversion to a revision stem. In 5 patients, multiple cerclage wires were placed around the shaft area, and in 2 patients, a cerclage wire was placed below the lesser trochanteric area. In 2 patients, the surgeon did not take any additional steps besides the administration of partial weight bearing. In one patient, the fracture was not recognized intraoperatively. This patient underwent revision surgery with cerclage wiring and periprosthetic locking plate fixation 7 days later. In another patient, dislocation of the cerclage wire with the loosening of the prosthesis occurred. This patient underwent revision arthroplasty 6 weeks postoperatively. While 22 overall patients with infections were found (4.6%), two patients with infection were recorded in the fracture group (5.9%). In one patient, infection could be controlled by debridement, irrigation, and conversion to total hip arthroplasty. In the other case, the patient rejected surgery. An intraarticular drain was placed at the bedside. The patient later died due to renal failure. In another case, the patient died postoperatively due to respiratory failure while in the intensive care unit (Table 1; Fig. 3).

**Table 1:** Characteristics of IPF

Classification		
Vancouver	Type A	25/34
	Type B	8/34
	Type C	1/34
Canal type (Dorr)		
A		4/34
B		15/34
C		15/34
Canal Flare Index		
Champagne flute		0
Normal		22/34
Stovepipe		12/34
Follow-up radiographs		
Union		8/16
No union		8/16
Salvage treatment		
Figure of 8 cerclage		23/34
Cerclage shaft		5/34
Lesser trochanter cerclage		2/34
Conversion to revision stem		1/34
Conservative		2/34
Not recognized		1/34
Complications		
Death within same hospital stay		2
Infection		2
Revision due to implant failure		1

Characteristics of 34 intraoperative periprosthetic fractures (IPF)

**Fig. 4:**
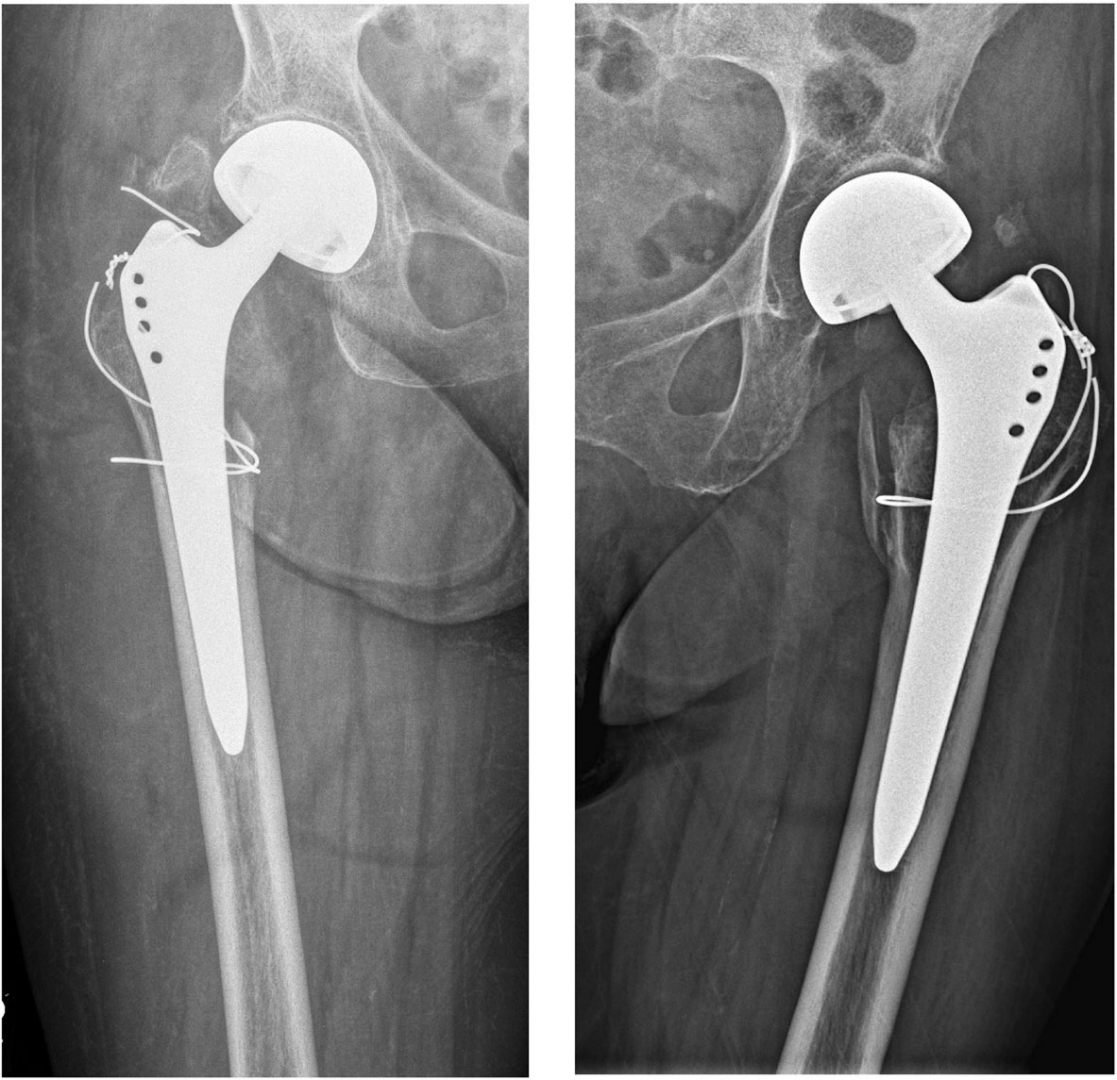
Failure of cerclage wire fixation in follow-up radiographs (a + b). Failure of cerclage wire and significant migration of trochanteric fragment with corresponding non-union

Of the 34 patients with IPFs, 10 follow-up radiographs could be found in our database that were at least 3 months after the surgery. With these radiographs, fracture union could be determined. We found 5 of 10 (50%) fractures to have reached union at least 3 months postoperatively; 4 of these were Vancouver type B fractures, and 1 was a Vancouver type A fracture. Five fractures (50%), which were all identified as Vancouver type A fractures, did not show any signs of union. In 3 of these patients, failure of the cerclage wire also occurred (Fig. 4).

There were no significant differences between the fracture and non-fracture groups regarding age, laterality, body mass index (BMI), pre-existing comorbidities, types of anesthesia, surgical approach, or the patients’ ASA score. Sex was not a significant risk factor (*p* = .084). However, intraoperative fractures were more likely to occur in female patients (OR 2.30; 95% CI .872–6.079).

The Dorr type C femur (*p* = .004) was found to be a significant risk factor for the development of IPF. The OR of sustaining an intraoperative fracture with a Dorr type C femur was 3.176 (95% CI 1.552–6.496) when compared with the Dorr type A or B femur. CFI was not an independent risk factor (*p* = .299). However, the OR of sustaining a fracture with an SP femur was 1.749 (95% CI .823–3.713) when compared with an N or CF femur.

Neither the surgeon’s experience nor the timing of surgery (daytime vs. on-call duty) yielded a significant difference. However, IPFs were more likely when surgery was performed by a junior surgeon when compared with a senior surgeon (OR 1.204; 95% CI .596–2.432) and more likely when surgery was performed during on-call duty when compared with daytime surgery (OR 1.471; 95% CI .711–3.046). The duration of surgery was significantly higher in the fracture group (94 min vs. 74 min; *p* < .001).

Cementation was not a significant risk factor (*p* = .104). However, the occurrence of IPF was more likely with uncemented arthroplasty when compared with cemented arthroplasty (OR 5.018; 95% CI .674–37.384) (Table 2).

**Table 2:** Patient demographics with or without intraoperative periprosthetic fracture

Characteristics	Intraopertive femoral fracture, *n* = 34	No intraoperative femoral fracture, *n* = 447	*p* value	Test used
Age, years	84 (SD 5.37)	83.6 (SD 7.63)	.704	Mann-Whitney *U*
BMI	24.84 (SD 4.46)	23.98 (SD 3.90)	.268	Mann-Whitney *U*
Duration of surgery, min	93.50 (SD 29.41)	73.99 (SD 19.86)	< .001	Mann-Whitney *U*
Duration of hospital stay, days	11.82 (SD 6.70)	11.57 (SD 9.45)	.689	Mann-Whitney *U*
Gender				
Male	5 (14.7%)	127 (28.4%)		
Female	29 (85.3%)	320 (71.6%)	.084	Chi-square
Laterality				
Right	13 (38.2%)	240 (53.7%)		
Left	21 (61.8%)	207 (46.3%)	.082	Chi-square
ASA score				
1	1 (3.0%)	1 (0.2%)		
2	10 (30.3%)	111 (25.1%)		
3	20 (60.6%)	296 (66.8%)		
4	2 (6.1%)	35 (7.9%)	0.192	Fisher-Freeman-Halton exact test
Surgical approach				
Bauer	21 (61.8%)	293 (65.5%)		
Watson-Jones	13 (38.2%)	152 (34.0%)		
Harding	0.00	2 (0.4%)	.748	Fisher-Freeman-Halton exact test
Surgeon’s experience				
Junior surgeons	15 (44.1%)	177 (39.6%)		
Senior surgeon	19 (55.9%)	270 (60.4%)	.604	Chi-square
Timing of surgery				
Daytime	12 (35.3%)	199 (44.5%)		
On-call duty	22 (64.7%)	248 (55.5%)	.296	Chi-square
Cementation				
Yes	1 (2.9%)	59 (13.2%)		
No	33 (97.1%)	388 (86.8%)	.104	Fisher’s exact test
Dorr classification				
A	4 (11.8%)	97 (21.7%)		
B	15 (44.1%)	261 (58.4%)		
C	15 (44.1%)	89 (19.9%)	.004	Chi-square
Canal Flare Index				
Champagne flute	0.00	13 (2.9%)		
Normal	23 (67.6%)	338 (75.6%)		
Stovepipe	11 (32.4%)	96 (21.5%)	.299	Fisher-Freeman-Halton exact test

Investigation and comparison of different demographics and characteristics in the fracture (*n* = 34) and non-fracture (*n* = 447) group. Statistical tests were used as appropriate. *P* values of < .05 indicate statistical significance

For the assessment of postoperative mobility, we received 17 responses (return 50%) by returned questionnaires or a subsequent telephone interview. The respondents were either relatives or the patients themselves. After completion of the questionnaire and telephone interviews, we were able to identify 12 matching partners. The average preoperative mobility was 2.50 on the scale according to NHFD, whereas the average postoperative mobility in the fracture and the non-fracture group was 3.33 and 2.92, respectively, accounting for a score difference of .41 points.

## Discussion

Fractures of the proximal femur are a major health problem in elderly persons. Although surgery is usually successful, few people recover fully, and there is a significant impact on their quality of life. Many become less independent and are at increased risk of becoming institutionalized following surgery [30, 31].

IPF is a well-described complication following total hip arthroplasty and hemiarthroplasty. While the majority of studies have investigated this complication in total hip arthroplasty, there are a number of studies that have focused on hemiarthroplasty [9, 12, 13, 19–21]. Among these, the primary intention has been the comparison between cemented and uncemented techniques. In randomized controlled trials, Parker et al. [9] and Taylor et al. [19] have shown the fracture incidence to be significantly higher with uncemented hemiarthroplasty. The overall fracture rate of 7.1%, as well as the isolated fracture rate of 7.4% for uncemented arthroplasty as determined by this study, was consistent with the current literature [20, 21], especially when considering that 87.5% of procedures were cementless.

We have traditionally preferred cementless fixation of the femoral component in both hemiarthroplasty and total arthroplasty of the hip joint. Until recently, the Zweymüller Alloclassic stem was the implant of choice [32]. Indeed, in 407 out of 481 procedures (84.6%), this very type of implant was used. This homogenous study population allowed us to retrospectively focus on risk factors in a widely unbiased fashion. To our knowledge, this is a study design, which has been adopted by only two other studies [20, 21]. Both studies used smaller population sizes (278 and 271, respectively) as well as heterogeneity of implant types. Moreover, these studies were both conducted in Singapore. To our knowledge, there has been no such European or American study yet.

The Zweymüller Alloclassic stem is a cementless tapered straight (rectangular) titanium femoral component. Its primary fixation is achieved by diaphyseal press-fit and secondary stability by bone ongrowth onto the grit-blasted surface [33]. This stem design allows for good anchorage along the entire prosthesis as well as for rotational stability independent of the patient’s individual form of the femur [34]. Indeed, in a study by Suckel et al. [35], the Zweymüller Alloclassic stem showed a survival rate of 98% at 17-year follow-up. However, due to its prominence in the proximal area, preparation of the dorsal corticalis in the greater trochanter area with the rongeur and the box chisel is mandatory to accommodate the prosthesis in a neutral position. This leads to a decrease in bone stock in the trochanteric area, which in turn may lead to a higher likelihood of IPF when compared with other stem designs.

Minimizing the risk of IPF may be associated with the identification of potential risk factors [23]. Contrary to comparable studies, uncemented hemiarthroplasty was not a significant risk factor (*p* = .104) in this study, although this can be ascribed to the relatively small number of cemented procedures (60/481 = 12.5%) within our study population, with only a single IPF occurring during cemented arthroplasty. If a larger group of cemented arthroplasties had been available, this would very probably have resulted in a significant correlation.

The consideration of proximal femur geometry is of vital importance in the decision-making process. We found that patients with a Dorr type C proximal femur were at significant risk of sustaining an IPF (*p* = .004). A decrease in the cortical index (CI) comes with age and is especially seen in older women [36]. These changes lead to a reduction in bone strength and thus greatly reduce the risk of fracture [37]. In concordance with this, female patients were at increased risk of sustaining intraoperative fractures within our study population, although this finding was not statistically significant (OR 2.30; *p* = .084).

When comparing hemiarthroplasty with total hip arthroplasty, the operative time and blood loss are significantly lower with hemiarthroplasty [38, 39]. Since it is technically not too demanding, this procedure is frequently performed by junior surgeons as part of their residency as well as during on-call duty. In the literature, there are little data scrutinizing the effects of surgeons’ experience on surgery-related complications. Additionally, there are little data investigating whether surgeries performed during on-call duty lead to higher complication rates. In this study, we found that procedures with IPFs were more likely to have been performed by a junior surgeon and more likely to have been performed during call duty when compared with procedures without intraoperative fractures. However, neither finding was significant (*p* = .592 and *p* = .371, respectively). Similarly, Schliemann B et al. [29] found trends but no significant differences when comparing the complication rates of bipolar hemiarthroplasty when performed by junior surgeons as opposed to senior surgeons (9.56% vs. 6.25%; *p* = .248) and when performed during on-call duty as opposed to daytime surgery (10.91% vs. 6.89%; *p* = .297). A recent study by Spaans et al. [40] on hip hemiarthroplasties did not find a significant difference with regard to the surgeon’s experience.

The postoperative mobility of patients undergoing hip hemiarthroplasty is difficult to assess, since the majority of patients are either elderly, suffer from dementia, or live in care homes and do not appear for follow-up visits by themselves. Furthermore, a high percentage of patients will have died within a few years after surgery. To assess the mobility of patients who sustained an intraoperative femoral fracture when compared with patients who did not, we were able to show that mobility was somewhat worse in the fracture group (difference of .41 points). Due to the methodical weakness of the approach used (variable time since surgery, low sample size), a statistical analysis was difficult. Given these circumstances, this result is difficult to interpret. Comparable studies have shown mixed results. In a recent study by Brun et al. [41], patients who sustained a periprosthetic fracture postoperatively had a significantly poorer outcome on the Oxford Hip Score, Pain VAS, Satisfaction VAS, and EQ-5D when compared with total hip replacements without fracture. In contrast, in a study by Liu et al. [42], patients with a postoperative fracture of the trochanter following total hip arthroplasty showed no impaired function and no symptoms after an average follow-up of 40 months. However, comparable to our study, the fracture group was small in both studies (26 and 11, respectively).

The surgeon must have a high amount of suspicion for iatrogenic fractures. During insertion, a sudden change of resistance is highly indicative of a fracture. Intraoperative C-arm images may help diagnose intraoperative fracture if a concern is raised. Before closure, the stability of the implant must be ensured [43]. Treatment depends on the location/classification of the IPF. Generally, cerclage wiring has been the treatment of choice in our clinic. In Vancouver type A fractures, we preferred a double loop technique in a figure of 8 manner (Fig. 4). In Vancouver type B fractures, we have usually placed multiple single loops around the shaft area containing the prosthesis. In particular, fractures of the greater trochanter seem to pose a challenge. It is known from previous studies that non-union of the greater trochanter fracture may result in impaired function or dysfunction of the abductor lever arm, causing pain and a Trendelenburg gait pattern [44]. In 5 of 6 Vancouver A fractures with available postoperative radiographs, there was no sign of a bony union or significant migration of the trochanter. Moreover, the cerclage wire failed in 3 patients. While the double loop technique is one of the treatments most often described in the literature, various alternatives can be deployed for trochanteric fixation. Among these, trochanteric claw plates, the greater trochanter reattachment device (GTRD = claw plate + cables) [45], tension band wiring [46], or Ethibond suture fixation [47] have been described. However, these techniques have mostly been used in the context of greater trochanteric osteotomies or unstable intertrochanteric fractures rather than IPFs of the greater trochanter. In terms of stability and union rates, cable grip systems have outperformed cables as well as wires [48–50]. In contrast, in a study investigating trochanteric fractures following total hip arthroplasty, Pritchett et al. [51] concluded that trochanteric fractures need no fixation unless there is severe dislocation or instability of the prosthesis, severe limp or pain or wide dislocation of the trochanteric fragment. Consistent with this finding, Rüdiger et al. [52] found that in a collective of 484 patients undergoing total hip replacement, 7 of 8 fractures that had occurred intraoperatively healed after conservative treatment. However, the Western Ontario and McMaster Universities Osteoarthritis Index (WOMAC) functional score was worse when compared with patients in the non-fracture group. One study investigating early postoperative periprosthetic fractures [53] found that 9 of 11 patients who sustained a Vancouver A(G) fracture failed operative treatment. Considering these mixed results and the poor union rate in this study, the treatment strategy for type A fractures should be scrutinized. A longitudinal controlled study comparing different fixation techniques with conservative treatment would be mandatory to determine which option yields better results.

This study is limited by its retrospective nature. Although the overall number of patients was high, the actual fracture group was relatively small. This eventually compromised the statistical analysis. Had there been a larger fracture group, additional significant risk factors might have been found. In particular, the mobility analysis suffered from a low number of recruited patients. Similarly, follow-up radiographs were rare, leaving it unclear as to whether the poor union rate found for type A fractures treated with cerclage wiring was a random finding. Furthermore, the method of assessing mobility was based on a subjective assessment from relatives or the patients themselves rather than on a clinical examination of the patients. To assess mobility and functionality in a more reliable fashion, a prospective controlled study design would be mandatory, following up patients at different intervals to test function and gait, as well as inquiring about their mobility and quality of life. However, the recent trend in our clinic has increasingly been towards cemented hemiarthroplasty, meaning that a significant drop in the intraoperative fracture rate is to be expected. Thus, acquiring a considerable number of patients in the fracture group would probably only be possible within a high-powered multi-center study.

The strength of this study lies in the large number of patients being reviewed. To our knowledge, this is the single largest study reviewing the risk factors of IPFs during bipolar hemiarthroplasty. Another strength lies in its homogenous study population. In nearly 85% of the procedures reviewed, the uncemented Zweymüller Alloclassic shaft was used, allowing for a largely unbiased analysis of risk factors. Having identified the Dorr canal type C as a significant risk factor, surgeons should always be aware of this canal type in the preoperative decision-making process. To our knowledge, this is the first study to try to compare postoperative mobility between patients who have sustained an IPF during hemiarthroplasty and patients who have not. We were able to show that there is a trend towards worse mobility for patients sustaining an IPF, which could translate into an increased risk of secondary complications.

## Conclusions

IPF is a serious complication that is not infrequently encountered with uncemented hemiarthroplasty. The identification of risk factors preoperatively, especially consideration of femur shape, is crucial and should be incorporated into the decision-making process.

## Data Availability

The datasets used and/or analyzed during the current study are available from the corresponding author upon reasonable request.

## Abbreviations

IPF: Intraoperative periprosthetic fracture(s)
ASA: American Society of Anesthesoiologists
CI: Cortical Thickness Index
CFI: Canal Flare Index
SP: Stovepipe (canal)
N: Normal (canal)
CF: Champagne flute (canal)
TIVA: Total intravenous anesthesia
NHFD: National Hip Fracture Database
OR: Odds ratio
CI: Confidence interval
BMI: Body mass index
WOMAC: Western Ontario and McMaster Universities Osteoarthritis Index
